# The bacterial communities of *Drosophila suzukii* collected from undamaged cherries

**DOI:** 10.7717/peerj.474

**Published:** 2014-07-22

**Authors:** James Angus Chandler, Pamela M. James, Guillaume Jospin, Jenna M. Lang

**Affiliations:** 1 Department of Evolution and Ecology and Center for Population Biology, University of California, Davis, CA, United States of America; 2 Department of Medical Microbiology and Immunology, University of California Davis Genome Center, University of California, Davis, CA, United States of America

**Keywords:** Microbiome, Microbiota, Symbiosis, Host-microbe interaction, *Drosophila*

## Abstract

*Drosophila suzukii* is an introduced pest insect that feeds on undamaged, attached fruit. This diet is distinct from the fallen, discomposing fruits utilized by most other species of *Drosophila*. Since the bacterial microbiota of *Drosophila*, and of many other animals, is affected by diet, we hypothesized that the bacteria associated with *D. suzukii* are distinct from that of other *Drosophila*. Using 16S rDNA PCR and Illumina sequencing, we characterized the bacterial communities of larval and adult *D. suzukii* collected from undamaged, attached cherries in California, USA. We find that the bacterial communities associated with these samples of *D. suzukii* contain a high frequency of *Tatumella*. *Gluconobacter* and *Acetobacter*, two taxa with known associations with *Drosophila*, were also found, although at lower frequency than *Tatumella* in four of the five samples examined. Sampling *D. suzukii* from different locations and/or while feeding on different fruits is needed to determine the generality of the results determined by these samples. Nevertheless this is, to our knowledge, the first study characterizing the bacterial communities of this ecologically unique and economically important species of *Drosophila*.

## Introduction

*D. suzukii* is an introduced pest insect that has recently become established in both North America and Europe (Rota-Stabelli, Blaxter & Anfora, 2013). The economic impact of *D. suzukii* in fruit growing regions may be substantial (Bolda, Goodhue & Zalom, 2010). Unlike most species of *Drosophila*, *D. suzukii* has a serrated ovipositor that allows it to lay its eggs in undamaged fruit (Rota-Stabelli, Blaxter & Anfora, 2013). This is distinct from most other *Drosophila*, including the closest relatives of *D. suzukii*, which lack a serrated ovipositor and therefore lay eggs in fallen and damaged fruit (Ashburner, Golic & Hawley, 2004; Mitsui, Takahashi & Kimura, 2006; Rota-Stabelli, Blaxter & Anfora, 2013). Therefore, the diet of *D. suzukii* is different from that of most other species of *Drosophila*.

The microbial communities associated with natural *Drosophila* populations are well characterized (for a review see Broderick & Lemaitre, 2012). Most studies have focused on the bacterial communities of *Drosophila* that feed upon fallen fruit (Cox & Gilmore, 2007; Corby-Harris et al., 2007; Staubach et al., 2013; Wong, Chaston & Douglas, 2013), while others have looked at additional host diets, such as mushrooms, cacti, and flowers (Chandler et al., 2011). The yeast communities of various *Drosophila* species have also been investigated (Chandler, Eisen & Kopp, 2012), and the yeasts associated with *D. suzukii* feeding upon undamaged fruits have been characterized (Hamby et al., 2012). However, to our knowledge, the bacterial communities of *D. suzukii* have not been examined.

In *Drosophila*, both laboratory and natural studies have found that diet plays an important role in shaping bacterial communities (Chandler et al., 2011; Staubach et al., 2013; Sharon et al., 2010). Since *D. suzukii* consume a distinct diet compared to other *Drosophila*, we hypothesized that this may play a role in shaping their bacterial communities. We therefore characterized the bacterial communities of adult and larval *D. suzukii* collected from undamaged cherries.

## Materials and Methods

On June 28th 2012 at Wolfskill Experimental Orchard near the town of Winters, California, USA, adult Drosophilids were aspirated directly from attached cherries (cherry variety DPRU0327/PRUNUS/AVIUM/F 98 CAROON/C 1 52). No insecticides or fungicides were applied in this orchard during this growing season. No specific permits were required for the described field studies and site managers provided informed consent before collections took place. Collected Drosophilids were stored alive in autoclaved glass vials for transport to the University of California, Davis (UCD) where they were positively identified as *Drosophila suzukii* (24 males and 1 female). Intestines were dissected from the males under sterile conditions and randomly divided into three sets of eight intestines each. Total time between collection and dissection did not exceed four hours. Whole cherries that lacked any visible damage were collected from the same tree and placed in sterile plastic bags for transport to UCD. The cherries were macerated in the bags and the largest visible larvae were picked from the bags, externally washed in 70% ethanol, rinsed in sterile water, and divided into three sets of ten individuals each. Additional larvae were collected from the same cherries, washed and rinsed as described above, and then individually placed in yeast extract-peptone-dextrose (YEPD) plates (1% yeast extract, 2% peptone, and 2% glucose/dextrose). The larvae were allowed to migrate for 30–60 s and the resulting colonies were used in a complementary study (Dunitz et al., 2014). The larvae were then individually placed in plastic vials containing Bloomington *Drosophila* media and all eclosing adults were positively identified as *D. suzukii* (4 males and 6 females).

DNA extractions were performed on these larvae and the adult intestines as previously described (Chandler et al., 2011). Bacterial DNA was amplified by a two-step PCR targeting the 16S rDNA gene (V4 region) with primers 515F and 806R, designed to include Illumina adaptor and barcode sequences. Sequencing was performed on an Illumina MiSeq at the UC Davis Genomics Core Facility generating 150 basepair paired-end reads. Samples were multiplexed with dual barcode combinations and demultiplexed with a custom script. After demultiplexing, the six samples had between 71,131 and 1,388 raw paired-end sequences for a total of 279,046 paired-end sequences. Paired sequences were combined using FLASH (Magoč & Salzberg, 2011) with parameters of a minimum overlap of 20 base pairs and a maximum overlap of 120 base pairs. These parameters were chosen to accommodate the 150 base pair paired-end reads used here (Jeff Froula, pers. comm., 2013). Other parameters were left as default.

Merged sequences were quality checked using QIIME (Caporaso & Kuczynski et al., 2010b) and default settings (Bokulich et al., 2013). Using UCLUST (Edgar, 2010), the 267,204 quality-checked sequences were clustered into *de novo* OTUs at the 97% similarity threshold producing 3,518 OTUs. The most abundant sequence in each OTU was chosen as a representative sequence. The representative sequences for all OTUs are available in Data S1. These representative sequences were screened for chimeras using the PyNAST aligner (Caporaso et al., 2010a) and ChimeraSlayer (Haas et al., 2011). Any OTU containing only 1 sequence was removed thus removing 2,878 OTUs (and therefore 2,878 sequences).

Taxonomic assignments were generated by querying the representative sequences against the truncated SILVA SSU Reference Database Release 111 (Quast et al., 2013) using the Blastn algorithm (Altschul et al., 1990) (Data S2). Any OTU with a best hit to mitochondria, chloroplast, or *Wolbachia* was removed from further analysis. Two OTUs with low query coverage (<63 basepairs) within the SILVA database were removed. Since we are primarily interested in the bacterial microbiota, four Archaeal OTUs were also removed. One of the larval libraries contains less than 300 sequences (all others contain greater than 35,000; Table 1) and was removed from subsequent analyses, which also removed four OTUs that were unique to this library (totaling eight sequences). The final dataset consists of 617 OTUs containing 256,274 total sequences. The proportions of the six most abundant OTUs in each sample are given in Table 1. Further details, including information on the more rare OTUs, the singleton OTUs, and the removed larval library, are found in Data S3.

**Table 1:** Proportion of the most abundant OTUs in each sample of *D. suzukii.* OTUs are identified by their closest hit in the SILVA SSU Reference Database Release 111. Number of sequences is after all quality-control steps. L, larva; A, adult.

	L1	L2	A1	A2	A3
*Tatumella punctata*	0.991	0.989	0.309	0.990	0.800
*Gluconobacter cerinus*	0.001	<0.001	0.658	0.002	0.123
*Acetobacter cerevisiae*	<0.001	<0.001	0.021	<0.001	0.028
*Dyella sp.*	0	0	0	0	0.015
*Gluconobacter oxydans*	<0.001	<0.001	0.001	0	0.009
*Orbus sp.*	<0.001	0	0	0	0.008
All other taxa	0.001	0.001	0.008	0.001	0.011
Total number of sequences in sample	50,701	65,346	55,426	44,545	40,256

**Figure 1:**
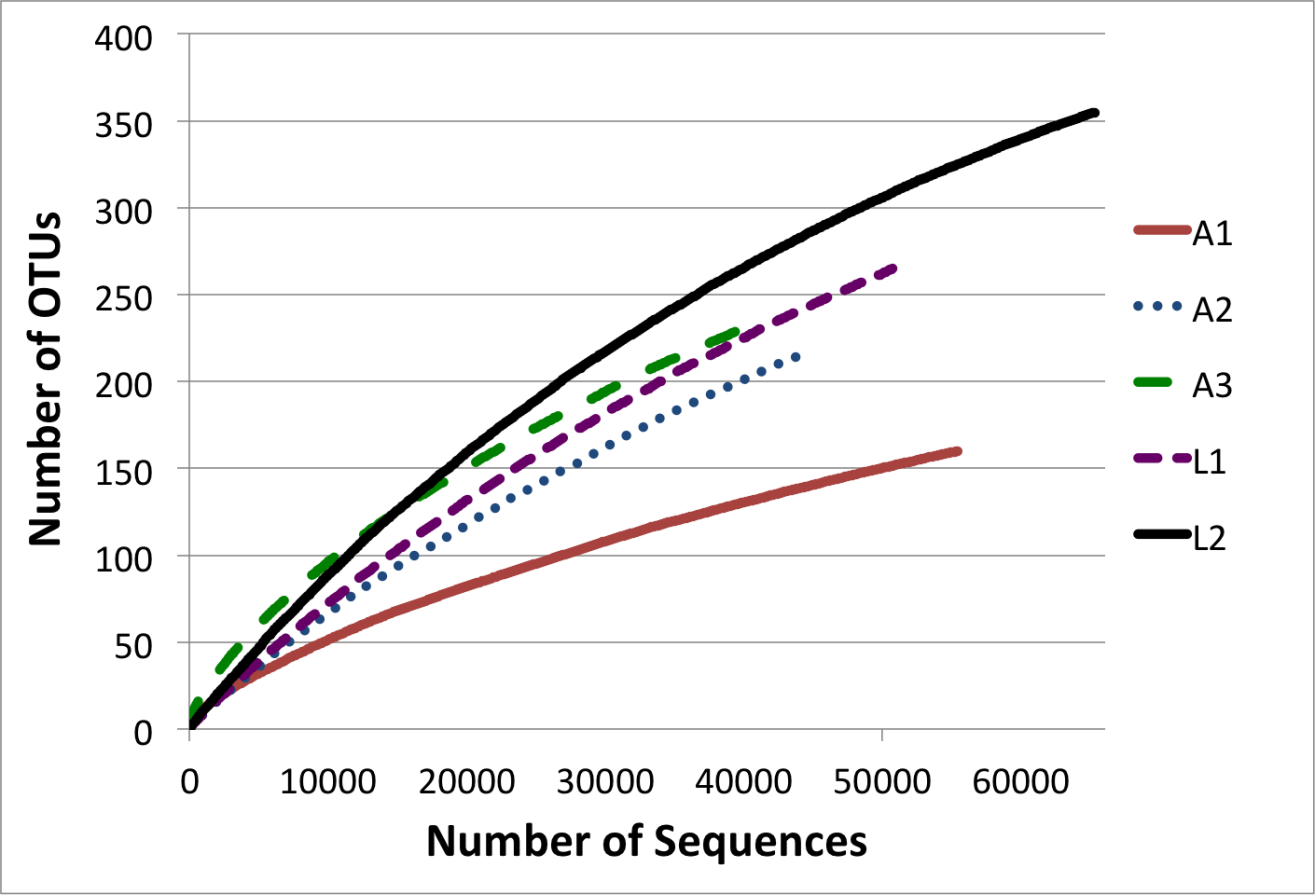
Rarefaction analysis of observed richness of the *D. suzukii* bacterial communities.

**Figure 2:**
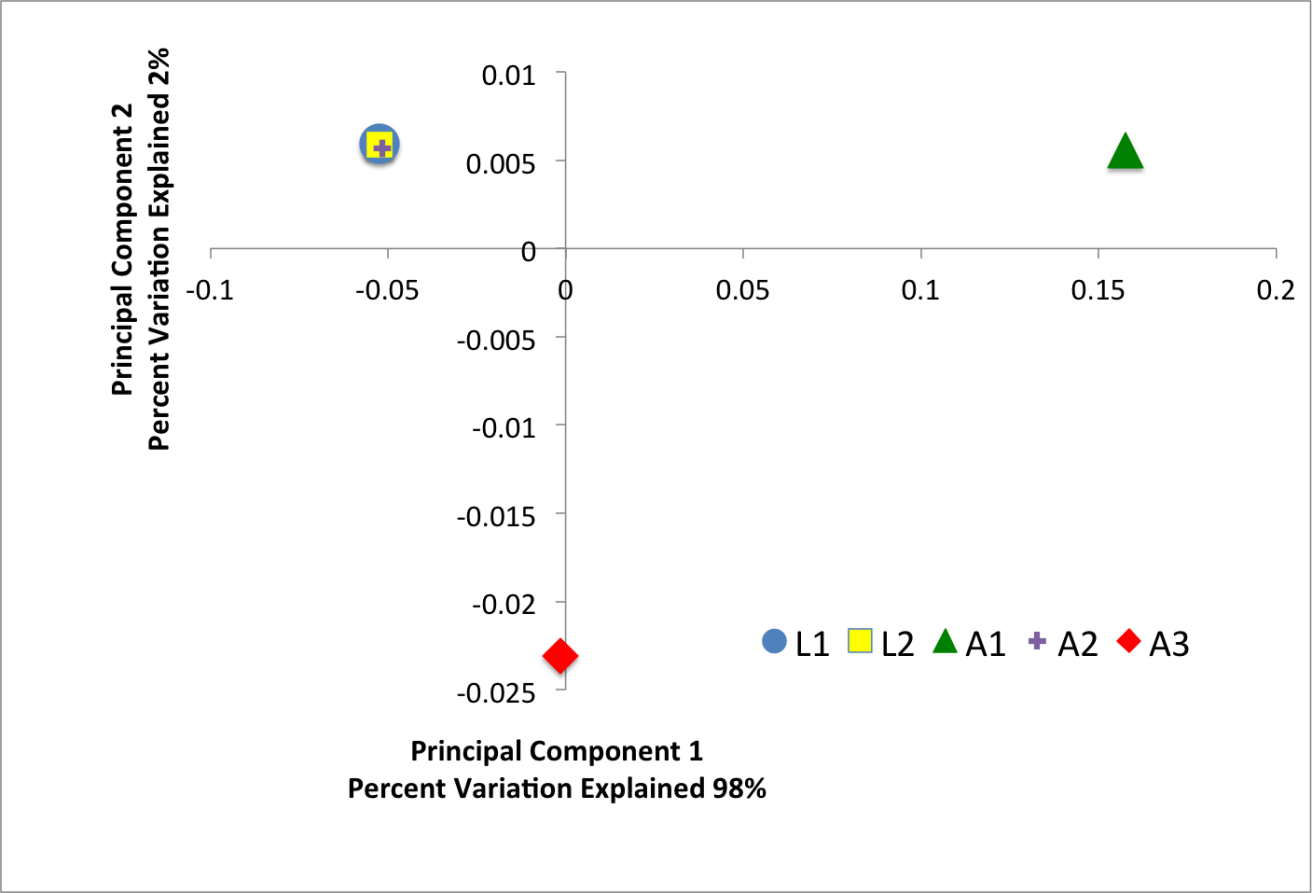
Weighted UniFrac principle coordinate analysis of the *D. suzukii* bacterial communities.

Alpha diversity was determined in QIIME by rarefying each sample to 35,000 sequences and taking the average of 100 iterations of rarefication (Table 2). Rarefaction curves of the observed OTUs were made in mothur using 100 iterations of the UCLUST generated OTUs (Schloss et al., 2009) (Fig. 1). Beta diversity was determined using weighted UniFrac (Lozupone & Knight, 2005) after aligning the representative sequencing using PyNAST (Caporaso et al., 2010a), building a phylogenetic tree using FastTree (Price, Dehal & Arkin, 2010), and rarifying each sample to 35,000 sequences in QIIME (Fig. 2).

Demultiplexed sequenced reads are available through NCBI’s Sequence Read Archive (SRA) under project number SRX391503.

## Results and Discussion

We characterized the bacterial communities of adult and larval *Drosophila suzukii* collected from undamaged, attached cherries. Three adult samples, each containing eight dissected male intestines, and two samples of larvae, each containing ten whole, externally sterilized individuals of unknown sex, are included in this analysis. 16S rDNA PCR and Illumina sequencing generated over 40,000 reads per sample (Table 1). Operational taxonomic units (OTUs) were formed by clustering sequences at the 97% similarity cutoff. Taxonomic assignments were generated by querying the representative sequence of each OTU against the truncated SILVA SSU Reference Database Release 111 using the Blastn algorithm (Data S2).

We find that the microbiota of both of the larval samples and adult sample A2 are composed of at least 99% *Tatumella*, and the remaining two adult samples contain 31% and 80% *Tatumella* (Table 1) (the larval sample that was excluded from formal analysis due to its extremely small library size was composed of 83% *Tatumella* [Data S3]). *Tatumella* is an Enterobacteriaceae that has been linked to both human and plant infections (Costa, Mendes & Ribeiro, 2008; Marin-Cevada et al., 2010). *Tatumella punctata,* the nearest hit to the largest *Tatumella* OTU identified in this study, was originally isolated from oranges (Kageyama et al., 1992). Although this genus is not considered a common *Drosophila* associate (Broderick & Lemaitre, 2012), it was recovered from *D. melanogaster* at an apple farm in New York, USA (Wong, Chaston & Douglas, 2013). Recently, several species previously classified as *Pantoea* have been transferred into *Tatumella* (Brady et al., 2010). Given that these species of *Pantoea* have been reported in *Drosophila* (Wong, Chaston & Douglas, 2013), perhaps *Tatumella* is a more common *Drosophila* associate than currently recognized. Nevertheless, *Tatumella* is the dominant bacteria associated with these samples of *D. suzukii*, while being absent, or at minimal levels, with other species of *Drosophila*. Sampling *D. suzukii* from different locations and/or while feeding on different fruits is needed to determine the ubiquity of the *D. suzukii*/*Tatumella* association.

The next most abundant taxa are species of Acetobacteraceae, specifically *Gluconobacter* and *Acetobacter* (Table 1). These are found in all five samples, but are primarily associated with adult samples A1 and A3. The Acetobacteraceae are commonly found associated with natural *Drosophila* populations (Chandler et al., 2011; Staubach et al., 2013; Wong, Chaston & Douglas, 2013; Corby-Harris et al., 2007; Cox & Gilmore, 2007). A minor, but notable, component to the bacterial community of adult sample A3 is a Gammaproteobacteria in the *Orbus* genus (0.8% of total community in A3). *Orbus* was the most common genus in a global survey of *Drosophilid* species (Chandler et al., 2011), but has not been recovered in most other studies of *Drosophila*-associated bacteria (Broderick & Lemaitre, 2012). The reasons for this are unclear, although it has been found at low frequencies in naturally collected fruit-feeding *D. melanogaster* and *D. simulans* (Staubach et al., 2013).

It is well established that alpha diversity measurements in 16S-based studies are affected by amplicon length, primer selection, alignment method, and quality control procedures (Schloss, 2010; Bokulich et al., 2013; Youssef et al., 2009). Furthermore, differences in sample collection and preparation can affect perceived bacterial diversity. For example, studies that examine whole bodies (Staubach et al., 2013; Wong, Chaston & Douglas, 2013) may have artificially high diversity compared to those using dissected intestines (such as was done here for the adult samples). Indeed, in laboratory raised flies, dissected intestines have slightly lower observed and Chao richness than whole bodies (Chandler et al., 2011). Furthermore, since transit time through the *Drosophila* intestine can be as low as 50 min (Wong et al., 2008), undue time between collection and sample preparation can affect diversity measurements as the individuals purge their intestinal contents. Because of these caveats, it is difficult to compare results of previous studies to those generated here (Fig. 1 and Table 2).

**Table 2:** Alpha diversity calculations for each sample of *D. suzukii.*

	L1	L2	A1	A2	A3
Observed OTUs	204.12	240.86	120.61	182.24	213.08
Observed OTUs-SD	5.53	8.78	5.35	4.61	4.59
Chao	475.54	505.76	272.45	448.24	464.91
Chao-SD	55.26	52.50	47.86	46.28	49.74
Shannon diversity	0.13	0.16	1.18	0.14	1.22
Shannon-SD	0.0038	0.0061	0.0048	0.0032	0.0044

**Notes.**

SD Standard deviation L larva A adult

Weighted UniFrac analysis (a phylogenetically-informed beta-diversity metric that takes into account between-sample frequency differences) finds the two samples of *D. suzukii* larvae harbor similar bacterial communities, while the three samples of *D. suzukii* adults each have a distinct community (Fig. 2). The same pattern was found in a weighted UniFrac analysis that did not exclude singleton OTUs (data not shown). Furthermore, the observed OTUs, Chao richness, and Shannon diversity are very similar for both larval samples, whereas the adult samples exhibit much higher between sample variability in these three indices (Table 2). It should be noted that a consequence of our pooling method means that it cannot be determined if this variability is the result of a single individual with a highly different bacterial community or if multiple individuals, each with the same community, were pooled together by chance. Furthermore, since whole larvae were used it cannot be determined if non-intestinal bacteria, for example in the trachea or salivary glands, are obscuring potential variability of the larval intestinal microbiota.

One explanation for the differences in variability between larval and adult samples is that larvae are confined to the fruit that they were laid into, while adults can travel to other surfaces where they can acquire different bacteria. This result informs other studies of *Drosophila*, and insect-microbe studies in general, many of which characterize only a single sample from each population under investigation. The variability of adult samples described here indicates that, despite pooling multiple individuals, a single sample may not provide an accurate representation of the microbiota associated with that population.

## Conclusions

In this study, we find that *Drosophila suzukii* larvae and adults harbor simple bacterial communities that are mostly dominated by *Tatumella*. As *D. suzukii* is a generalist feeder that has been introduced to many areas of North America and Europe, sampling *D. suzukii* from different locations and/or while feeding on different fruits is needed to determine the ubiquity of the *D. suzukii*/*Tatumella* association. Nevertheless, given the distinct food source of *D. suzukii* (relative to most *Drosophila* species), the potential role of *Tatumella* (or other, yet to be identified *D. suzukii*-associated bacteria) on host fitness or physiology is intriguing. In particular, the draft genome of the most abundant *Tatumella* strain associated with this population of *D. suzukii* is available (Dunitz et al., 2014) and analysis of this genome may reveal the metabolic potential of the microbiota to supplement the *D. suzukii* diet with nutrients that are scarce on unfallen fruit. Furthermore, by inoculating *D. suzukii* with defined bacterial communities under controlled dietary conditions, future experimental work can explicitly reveal the microbiota’s role in host biology. In summary, by characterizing the bacterial microbiota of these samples of *D. suzukii*, this study is the initial step in the investigation of the interplay between diet and bacteria in this interesting and economically important host-microbe system.

## Supplemental Information

10.7717/peerj.474/supp-1 Data S1 Representative sequences Fasta file containing the representative sequence of all 3,518 OTUs.

10.7717/peerj.474/supp-2 Data S2 Blastn results Results generated by querying the representative sequences of each OTU against the SILVA SSU Reference Database Release 111 using the blastn algorithm.

10.7717/peerj.474/supp-3 Data S3 Taxonomy counts Spreadsheet describing the abundance of each OTU in each sample. OTUs removed from the final analysis are indicated.

## Additional Information and Declarations

### DNA Deposition

The following information was supplied regarding the deposition of DNA sequences: NCBI’s Sequence Read Archive, Project Number SRX391503
